# Practice and Knowledge on Type 2 Diabetes Mellitus Risk Factors Among Office Workers in Mwanza City, Tanzania

**DOI:** 10.24248/eahrj.v7i1.712

**Published:** 2023-07-12

**Authors:** Haruna Dika, Magdalena Deogratias, Daniel Byamungu, Karol Marwa, Anthony Kapesa, Stanley Mwita

**Affiliations:** a Department of Physiology, Catholic University of Health and Allied Sciences, Mwanza, Tanzania; b School of Pharmacy, Catholic University of Health and Allied Sciences, Mwanza, Tanzania; c Department of Pharmacology, Catholic University of Health and Allied Sciences, Mwanza, Tanzania; d Department of Community Medicine, Catholic University of Health and Allied Sciences, Mwanza, Tanzania

## Abstract

**Background:**

Type 2 diabetes mellitus (T2DM) mostly occurs in adults when the body becomes resistant to insulin. Genetic predisposition, age, an unhealthy diet, and a sedentary lifestyle are key factors leading to T2DM. Office workers are one of the populations at greatest risk of developing T2DM. This study assessed the level of knowledge and risk factors for T2DM among office workers in Mwanza City, Tanzania.

**Methods:**

A cross-sectional study was conducted among 309 office workers in public and private institutions in Mwanza City. A structured, pre-tested questionnaire was used to collect information from the participants. The coded data were analyzed using STATA Version 14. The associations between various risk factors for T2DM and knowledge on T2DM were determined using Chi-square or Fisher's exact tests.

**Results:**

The level of knowledge was poor in 41.1%, moderate in 31.1%, and good in 27.8% of the study participants. Family history of T2DM showed a significant association with knowledge score (P=.001). Only 63 (20.4%) of respondents reported eating a healthy diet. Among the study participants, 154 (49.8%) had poor diabetes prevention practices, 82 (26.5%) had moderate practices, and 73 (23.7%) had good practices.

**Conclusion:**

The majority of the office workers who participated in this study had limited knowledge regarding risk factors for T2DM and poor practices concerning the prevention of the disease. In order to reduce the burden of T2DM, there is a need for lifestyle modification, provision of education, and raising awareness about the risk factors of T2DM among office workers in Mwanza City.

## BACKGROUND

Diabetes mellitus (DM) is one of the endocrine disorders that affect the body's capability to produce or use insulin. It is a chronic metabolic disease in which a person experiences high blood sugar, either because the pancreas does not produce enough insulin (Type 1 DM) or because the body cells do not efficiently utilize or respond to the insulin that is produced (Type 2 DM).^1^ According to the 2021 International Diabetes Federation (IDF) report^2^, 537 million adults aged between 20 to 79 years worldwide, are living with DM. This number is predicted to rise to 643 million by 2030. About 81% of adults with DM live in low and middle income countries (LMICs). In Africa, DM was responsible for 416,000 deaths in 2021. The IDF also reported that 11.6% of deaths in 2021 among people under the age of 60 years in Tanzania were diabetes related.

Type 2 DM (T2DM) mostly occurs in adults when the body becomes resistant to insulin.^3^ Globally, economic growth and urbanization have led to an increasing burden of T2DM.^4^ The disease is well known as a serious public health concern with a substantial effect on human life. Complications caused by T2DM include diabetic neuropathy, cardiovascular disease, cerebrovascular disease, and peripheral vascular disease.^5–7^ Genetic predisposition, age, an unhealthy diet, and a sedentary lifestyle are key factors leading to T2DM.^8^ Thus, lifestyle changes, including maintaining a healthy body weight, consuming a healthy diet, staying physically active, exercising, not smoking, and drinking alcohol in moderation, could decrease the risk of T2DM.^9,10^

Prevention of T2DM is very important and can be attained through lifestyle interventions, particularly among populations at great risk such as office workers.^11^ Adequate knowledge is a major component in T2DM prevention. A previous study reported poor knowledge regarding T2DM in the general population.^12^ Knowledge is paramount in the fight against T2DM, as it can help people assess their risk and inspire them to take responsibility for their health.^13^ Most knowledge and practice studies in LMICs related to T2DM have focused on patients.^14–18^ However, there is a paucity of reports from the general community, particularly among office workers in Tanzania.

An approach targeting individuals at risk of T2DM with the aim of reducing concomitant risk factors in the community is necessary. Knowledge and practices as regards risk factors and prevention of any disease in the community augment the achievement of any disease control program.^19^ This study assessed the level of knowledge and practice regarding risk factors for T2DM among office workers in Mwanza City, Tanzania.

## METHODS

### Study Setting and Design

A cross-sectional study was conducted in May 2021. The study was done in public and private institutions in Mwanza City, which is located on the shore of Lake Victoria in the north-western part of Tanzania. According to the 2022 census, the city's population was estimated to be 1,104,521.^20^ Mwanza City was chosen as the study site because it is an urban area with a high prevalence of T2DM.^21^

### Study population

The target study population was office workers aged 18 years and above. Office workers who were known to be diabetic or had a health professional background were excluded from this study.

### Sample size and sampling procedure

The sample size for this study was calculated using the Kish Leslie formula.^22^ Using the prevalence of the general population with good knowledge of T2DM from the Kenya study, which was 27.2%^23^, the minimum sample size was determined to be 304.

Four offices in Mwanza City were purposively selected as sites for collecting data. These offices were the Tanzania Revenue Authority (TRA) regional office, the Cooperative and Rural Development Bank (CRDB), the National Bank of Commerce (NBC), and the Regional Commissioner's office. A convenient sampling technique was used to recruit office workers in these offices who voluntarily agreed to participate in this study.

### Data collection

Two research assistants conducted face to face interviews with the study participants. A structured, pre-tested questionnaire developed in English and translated into the local language (Kiswahili) was used to collect information from respondents. The questionnaire was adopted from previous studies and modified to suit the current study population and objectives.^24,25^ the questionnaire had two parts. The first part contained the respondent's demographic information, which included age, sex, marital status, level of education, and family history of diabetes. The second part included questions to assess knowledge, risk factors and practice of preventing T2DM. The knowledge scale required the respondents to rate each item as either “true”, “false”, or “don't know”. A score of 1 was given for each right answer and 0 for each wrong answer. To evaluate the knowledge, we included 8 correct and 7 incorrect responses regarding risk factors for T2DM. The correct responses given were having a family history of diabetes, smoking cigarettes, being physically inactive, being 45 years of age or older, drinking too much alcohol, eating foods rich in carbohydrates, taking excessive sugary drinks, and being obese or overweight. Incorrect responses included eating food rich in fruits and vegetables, contact with a diabetic patient, infection with a virus or bacteria, drinking too much water, taking foods with too much salt, exposure to radiation or chemicals, and having high blood pressure. Score ranges of 0–5, 6–10, and 11–15 were considered poor, moderate, and good knowledge, respectively.^25^

To examine the practice, we assessed participants' lifestyles that promote T2DM prevention. We asked questions on eating a healthy diet, consistent physical activity, avoiding excessive use of alcohol and cigarettes, and regular blood glucose checkups. A score of 1 was given for each “yes” answer and 0 for each “no” answer. Score ranges of 0-2, 3, and 4-5 were categorized as having poor, moderate, and good practice, respectively. These practice scores of the respondents' lifestyles were adopted and modified from a prior study.^26^

### Statistical Analysis

The coded data was entered into an Excel worksheet, cleaned, and then exported to STATA Version 14 for analysis. Categorical variables were described as frequencies and percentages. The associations between various factors and knowledge or practice on risk factors for T2DM were determined using Chi-square or Fisher's exact tests, where appropriate. The statistical significance level was set at *P<*.05.

### Ethical Consideration

This study was approved by the Catholic University of Health and Allied Sciences and Bugando Medical Centre's Joint Ethics and Research Review Committee (IRB No. 1828/2021). Permission to conduct interviews was obtained from the directors and administrators of the respective institutions. Before the interview, written informed consent was obtained from the participants who voluntarily agreed to participate in the study. To ensure confidentiality, no participant's name was recorded.

## RESULTS

### Social Demographic Characteristics of Study Participants

We enrolled 309 (164 females and 145 males) office workers. Slightly over half of the participants, 163 (52.8%) aged between 18 and 32 years. Over 60% of the participants had at least college education and did not have a family history of T2DM (Table 1).

**TABLE 1:** Socio-demographic Characteristics of Study Participants

Variable	Categories	Frequency	Percentage
Age group	18–32	163	52.8
	33–41	82	26.5
	42–50	42	13.6
	>50	22	7.1
Sex	Male	145	46.9
	Female	164	53.1
Marital status	Married	145	46.9
	Single	127	41.1
	Separated	23	7.4
	Widowed	14	4.5
Level of education	Secondary	110	35.6
	College and above	199	64.4
Family history of diabetes	Yes	117	37.9
	No	192	62.1

### Knowledge on Risk Factors for Diabetes

Percentage of participants with knowledge on risk factors for T2DM ranged from 23.3% for smoking to 68% for physical inactivity T2DM (Table 2). For instance, over 60% of participants knew that taking excessive sugary food, drinks and alcohol is a risk for developing T2DM. Over half of the interviewed office workers knew that having a family member with diabetes, obesity and overweigh place a person at risk of T2DM.

**TABLE 2:** Study Participants' Knowledge on Diabetes Risk Factors

Risk factors	Response	Frequency	Percentage
Family history of diabetes	True	170	55.0
	False	137	44.3
	Don't know	2	0.6
Physical inactivity	True	210	68.0
	False	97	31.4
	Don't know	2	0.6
Taking excessive sugary foods and drinks	True	205	66.3
	False	100	32.4
	Don't know	4	1.3
Drinking too much alcohol	True	197	63.8
	False	110	35.6
	Don't know	2	0.6
Obesity and overweight	True	183	59.2
	False	123	39.8
	Don't know	3	1.0
Smoking	True	72	23.3
	False	235	76.1
	Don't know	2	0.6
Taking too much carbohydrates	True	149	48.2
	False	156	50.5
	Don't know	4	1.3
Increasing age	True	150	48.5
	False	151	48.9
	Don't know	8	2.6

### Factors associated with knowledge regarding risk factors of diabetes

Only 27.8% of the participants demonstrated level of good knowledge on risk factors for diabetes. Of those, high proportion of participants (53.2%) had family history of diabetes. Other socio-demographic factors did not vary with the level of knowledge on risk factors for diabetes (Table 3).

**TABLE 3:** Factors Associated with Knowledge on Risk Factors of Diabetes

Variable	Knowledge			P value
	Good (N=86) %	Moderate (N=96) %	Poor (N=127) %	
Age group				
18–32	51.2	51.0	55.1	
33–41	24.4	28.1	26.8	.672
42–50	15.1	11.5	14.2	
>50	9.3	9.4	3.9	
Sex				
Male	52.3	46.9	43.3	.433
Female	47.7	53.1	56.7	
Marital status				
Single	38.4	41.7	42.5	
Married	50.0	41.7	48.8	.380
Separated	7.0	12.5	3.9	
Widowed	4.7	4.7	4.7	
Education level				
Secondary	27.9	37.5	39.4	.206
University	72.1	62.5	60.6	
Family history of diabetes				
Have family history	52.3	38.5	27.6	.001
No family history	47.7	61.5	72.4	

### Practice towards Prevention of Diabetes

Although majority of study participants (75.1%) do not smoke and 60.5% claim to perform physical activity, less than half (42.1%) check their blood glucose levels at once a year and 20.4% eat healthy diet (Table 4).

**TABLE 4:** Respondents' Healthy Style for Prevention of Diabetes

Variable	Response	Frequency	Percentage
Checking blood glucose level annually	Yes	130	42.1
	No	179	57.9
Smoke cigarette	Yes	77	24.9
	No	232	75.1
Eating healthy diet	Yes	63	20.4
	No	246	79.6
Drink alcohol	Yes	163	52.8
	No	146	47.2
Perform physical activity	Yes	187	60.5
	No	122	39.5

### Factors Associated with Practice towards Prevention of Diabetes

Generally, 154 (49.8%) study participants had poor diabetes prevention practices, 82 (26.5%) had moderate practices, and 73 (23.7%) had good practices. All socio-demographic characteristics did not associate with the practice of diabetes prevention (Table 5).

**TABLE 5:** Factors Associated with Practice of Preventing Diabetes

Variable	Practice			P value
	Good (N=73) %	Moderate (N=82) %	Poor (N=154) %	
Age group				
18–32	57.5	51.2	51.3	.816
33–41	20.5	26.8	29.2	
42–50	16.4	13.4	12.3	
>50	5.5	8.5	7.1	
Sex				
Male	45.2	41.5	50.6	.382
Female	54.8	58.5	49.4	
Marital status				
Single	38.4	47.6	39.0	.476
Married	52.1	39.0	48.7	
Separated	4.1	7.3	9.1	
Widowed	5.5	6.1	3.2	
Education level				
Secondary	45.2	26.8	35.7	.058
University	54.8	73.2	64.3	
Family history of diabetes				
Have family history	41.1	36.6	37.0	.807
No family history	58.9	63.4	63.0	

## DISCUSSION

The present study reports a low level of knowledge regarding risk factors for T2DM among office workers in Mwanza City. We found that only 27.8% of participants had good knowledge, which was positively associated with a family history of DM. Although about 60% of the participants engaged in physical activity, about 50% had poor practices towards T2DM prevention in general.

The small proportion of participants with good knowledge regarding risk factors for T2DM observed in our study is consistent with previous reports from other East African countries. For instance, Maina et al^22^ reported that only 27.2% of the community members in Kenya had good knowledge of TD2M. In the current study, a family history of T2DM was found to be associated with good level of knowledge, which is similar to the findings of several studies.^25,27,28^ A previous study revealed that having a family history of T2DM increases daily consumption of fruits and vegetables and participation in diabetes screening.^27^ However, non-diabetic people with a family history of T2DM who have low physical activity are at higher risk of developing diabetes because their beta cells are less likely to be able to compensate for the increased insulin resistance brought on by an increase in body mass index (BMI). ^29^

The majority of the current study participants knew that a family history of diabetes, physical inactivity, excessive intake of sugary foods and fluids, drinking too much alcohol, obesity, and being overweight are risk factors for T2DM. These findings are consistent with previous studies that were conducted in different populations.^25,30^ In the present study, only 23.3% of participants knew that smoking is a risk factor for T2DM. This is slightly lower than 28% reported in a Bangladesh study.^25^ Thus, more awareness campaigns about the harmful effects of smoking are necessary. These findings highlight very essential aspects of education for the community and health promotion regarding T2DM. Community knowledge about T2DM is a prerequisite for people to take action to control the disease. Knowledge affects their attitude and uptake of health services, including health education.^31^

In the current study, findings revealed that minority of the participants eat healthy foods. Office workers spend a large part of their time at work. Research suggests that the workplace environment can affect eating behaviors, leading to several health consequences, such as T2DM.^32^ The combination of a sedentary lifestyle and easy access to unhealthy foods increases the risk of being overweight or obese.^33^ Globally, obesity is a major risk factor for T2DM, and its prevalence is growing rapidly in sub-Saharan Africa.^34^ The previous study in Tanzania reported a significant association between obesity and glucose impairment in parts of the Kilimanjaro Region of Tanzania.^35^ Unless urgent actions are established to minimize unhealthy eating and sedentary lifestyles, the burden of T2DM is expected to continue growing.^36^ To encourage positive behavioural change, effective workplace health promotion that takes into account factors such as people, the environment, and policy is crucial.^37,38^

### Strength and Limitations

Our study is the first community based study among office workers assessing knowledge and practice regarding risk factors for T2DM in Tanzania. However, there are also limitations to our study. First, given the cross-sectional design, causal relationships cannot be drawn. Second, the study did not include qualitative methods, which would have explored more knowledge and practices among study respondents. As respondents were only taken from four offices within one city, it is difficult to generalize the findings as a base for the entire country of Tanzania. There is a need for future research to replicate our study with a more diverse sample of offices to increase the external validity of our results.

## CONCLUSION

The majority of the office workers who participated in this study had limited knowledge on risk factors for T2DM. Low proportion of participants demonstrated good practice for prevention of diabetes. The findings of this study suggest the need for lifestyle modification, the provision of education to the community, and raising their awareness about the risk factors for T2DM
